# Tracking neuronal motility in live murine retinal explants

**DOI:** 10.1016/j.xpro.2021.101008

**Published:** 2021-12-09

**Authors:** Nozie D. Aghaizu, Katherine M. Warre-Cornish, Martha R. Robinson, Robin R. Ali, Rachael A. Pearson

**Affiliations:** 1 University College London, Institute of Ophthalmology, London EC1V 9EL, UK

**Keywords:** Cell Biology, Developmental biology, Microscopy, Model Organisms, Neuroscience

## Abstract

The developing retina undergoes dynamic organizational changes involving significant intra-retinal motility of the encompassing cells. Here, we present a protocol for tracking retinal cell motility in live explanted mouse retinae. Although originally applied to rod and cone photoreceptors, this strategy is applicable to any fluorescently labeled cell in mouse retinae and other similar experimental retinal models. Careful tissue handling is critical for the successful acquisition of high-quality live imaging data. Further instructions for semi-automated *in silico* data handling are provided.

For complete details on the use and execution of this protocol, please refer to Aghaizu et al. (2021).

## Highlights

• Protocol for cell motility tracking in live murine retinal explants
• Fluorescent labelling of retinal cells using transgenic animals or by transfection
• Live-imaging set-up of murine retinae in a 2-photon imaging system
• *In silico* tracking and quantification of retinal cell motility

## Graphical abstract

**Figure:**
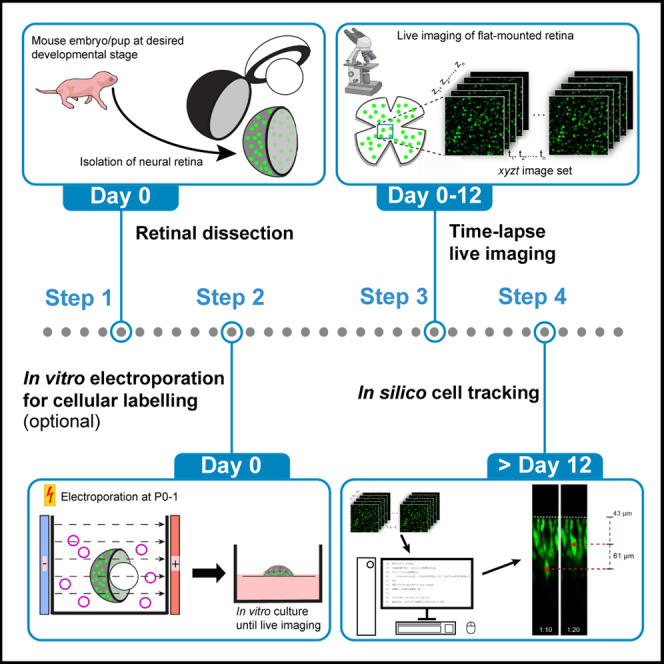

## Before you begin

This protocol was designed to track motions of fluorescently labeled retinal cells. In Aghaizu et al. (2021), cellular labeling was achieved either by using transgenic mouse lines expressing cell-type specific fluorescent reporters or by electroporation of acutely explanted retinae with an expression plasmid driving the expression of a fluorescent protein.

All mice in preparation of this manuscript were used according to the NC3R ARRIVE guidelines. Both male and female mice were used in this study without discrimination. All animal studies were carried out under the Animals (Scientific Procedures) Act 1986 under a project license PPL 70/8120 issued by the UK Government Home Office and conducted in accordance with protocols approved by the Animal Welfare and Ethics Committee of the UCL Institute of Ophthalmology. All animals were killed by cervical dislocation performed by trained personnel (approved under Schedule 1 as a method of humane killing). All efforts were made to minimize the number and suffering of animals used in these experiments.

Male and female mice were group housed in the animal facility at University College London on a standard 12-h light/dark cycle at the same light levels throughout the experimental period. Animals were kept in individually ventilated cages on animal grade wood chips and given access to nesting material and food and water ad libitum.

### Preparation of transgenic mice

**Timing: 2.5–4.5 weeks**

1. Cell-type specific reporter mice are used for the purpose of having fluorescently labeled rod or cone photoreceptors *in vivo.* Timed-matings need to be set up 2.5–4.5 weeks prior to retinal live imaging depending on the developmental stage of interest (typically between embryonic day (E) 16.5 and postnatal day (P) 10.5).
  a. For fluorescently labeled rod photoreceptors, time-mate *Nrl.GFP*^*+/+*^ mice (Akimoto et al., 2006). These mice are suitable for homozygotic breeding.
  b. For fluorescently labeled cone photoreceptors, time-mate *Chrnb4.EGFP* mice (Gong et al., 2003). These mice should be bred as hemizygotes.
  c. For Cre-expressing rod photoreceptors, time-mate *Nrl.Cre*^*+/−*^ mice (Brightman et al., 2016). These mice should be bred as hemizygotes.
2. Successful mating is typically accompanied by the presence of a vaginal plug, which marks embryonic day (E) 0.5 following overnight mouse pairing for 14–16 h. The gestation period in mice is usually 19–21 days (strain dependent). Subsequently isolate and live image retinae at desired time point (see ‘step-by-step method details’).

### Preparation of transfection-based cellular labelling

**Timing: 2.5–4.5 weeks including husbandry; 2.5 h for dissection, electroporation and culture setup**

**Figure 1:**
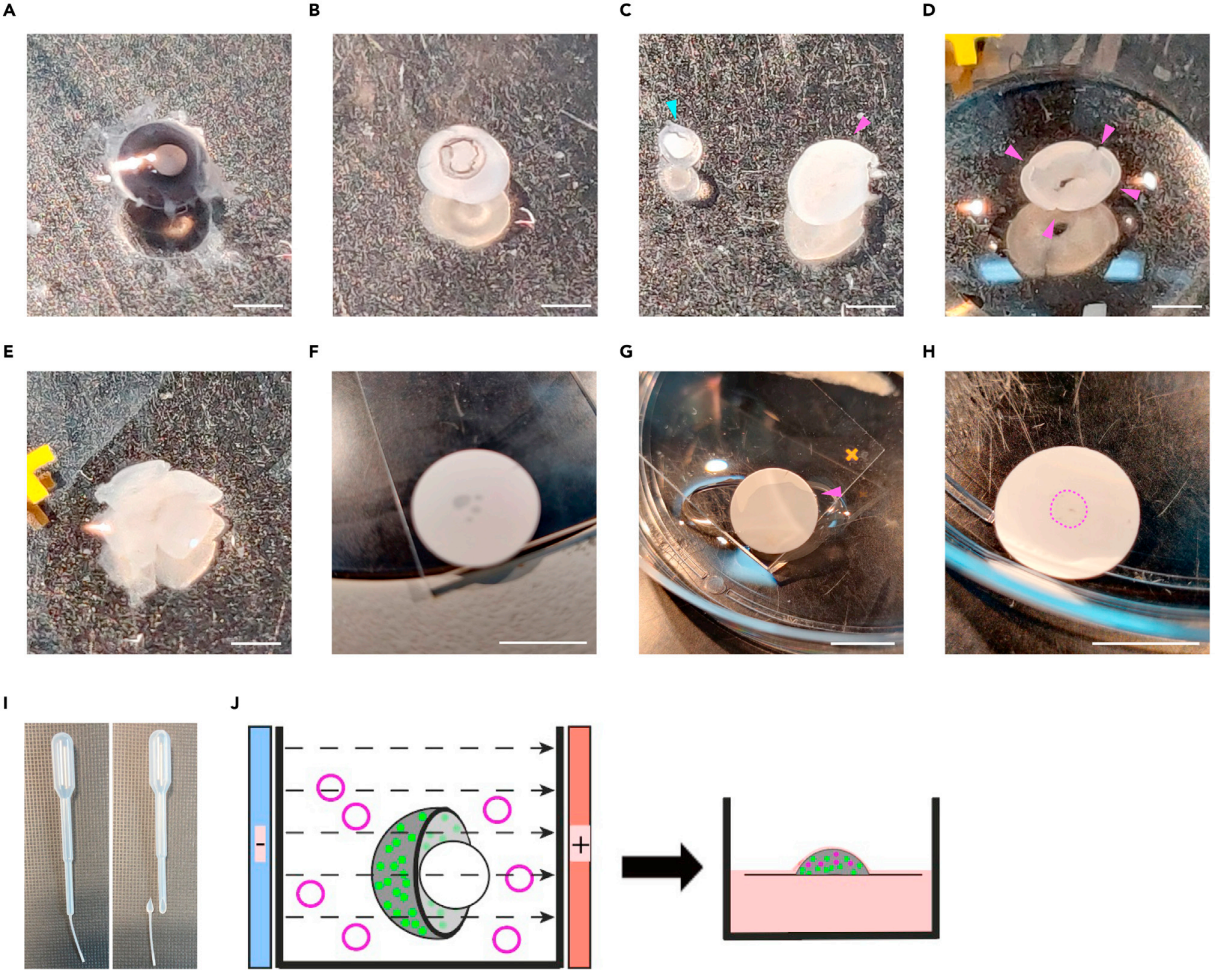
Retinal dissection and tissue handling (A) Enucleated eye from a P0 *Nrl.Cre*^*+/−*^ mouse. (B) Neural retina with lens left intact isolated from eyeball. (C) Lens (cyan arrowhead) separated from neural retina (magenta arrowhead). (D) Radial cuts (magenta arrowheads) applied to the neural retina. Tissue was also transferred onto the edge of the standard microscopy glass slide. (E) Neural retina flattened on a glass slide by unfolding the 4 ‘petals’. Excess liquid was removed (compared with (D)). (F) 0.45 μm MF-Millipore™ nitrocellulose membrane placed on top of flattened neural retina. (G) Membrane-mounted retina being transferred back into RPMI 1640 medium by dipping the edge of the glass slide into medium. The membrane will gradually become visibly hydrated during this step (magenta arrowhead). (H) Flat mounted neural retina (magenta dotted outline) on nitrocellulose membrane. Membrane was inverted compared to (G). (I) Plastic Pasteur pipette manually widened using sterilized scissors. (J) Schematic depicting optimal orientation of neural retina relative to electroporation electrodes. Magenta plastic circles symbolize plasmid used for electroporation. The subsequent step depicts the neural retinal culture setup, whereby the neural retina is placed on a floating Whatman® Nuclepore™ Track-Etched Membrane in a sitting drop. Scale bars, 1 mm (A–E), 10 mm (F–H).

3. Photoreceptor fluorescent labeling can also be achieved by introducing an expression plasmid of choice into the desired target retinal cells. We used a conditional strategy to limit fluorescent protein expression to rod photoreceptors only. This was achieved by electroporating a Cre-sensitive expression plasmid into acutely explanted retinae from *Nrl.Cre*^*+/−*^ mice as described below, although this can also be achieved by *in vivo* electroporation (Matsuda and Cepko, 2004) or AAV viral transduction (Aghaizu et al., 2021).
  a. Dissect out retinae from P0.5–1.5 mice obtained from timed mated pregnancies (see ‘preparation of transgenic mice’; (1.c)) in ice cold dissection medium (Figure 1A). Remove all non-neural retinal tissues (cornea, sclera, iris, choroid, optic nerve, retinal pigment epithelium, and ciliary body) with exception of the lens, which is left in place for increased tissue stability (Figure 1B).
    **CRITICAL:** To avoid contamination, dissection tools (#5 Dumont forceps, Vannas scissors) are to be sterilized with 70 % ethanol before dissection. Furthermore, aim to perform the majority of ‘Preparation steps no. 3’ in a sterile tissue culture hood.
    **CRITICAL:** Care should be taken to avoid damaging retinae during dissection as tissue disruptions introduced during this step tend to become amplified during *in vitro* culture. We found that dissected retinae are most gently transferred by using sterile plastic Pasteur pipettes (supplied with electroporation cuvettes) with a manually widened opening (Figure 1I).
  b. Transfer retinae to electroporation cuvette containing a 1 μg/μL plasmid DNA solution in PBS, prepared as described in ‘Materials and equipment’ (e.g., *shDync1h1/EGFP*; Aghaizu et al., 2021) and incubate at room temperature (18°C–21°C) for 5 min.
  c. Orientate retinae within the cuvette such that the apical and lens sides of the retina are aligned with the cuvette electrodes; for optimal electroporation efficiency the apical surface is to be juxtaposed to the anode (Figure 1J).
  d. Use a pulse generator to electroporate retinae with 5 × 30 V square pulses of 50 ms duration and with 950 ms intervals.
    **CRITICAL:** Avoid direct contact between electrodes and retinae during electroporation as this may result in substantial tissue damage.
  e. Allow retinae to recover in pre-equilibrated (37°C/5% CO_2_) dissection medium for 5 min.
  f. Transfer retinae to pre-equilibrated (37°C/5% CO_2_) culture medium for 5 min.
  g. For *in vitro* retinal explant cultures, transfer up to 3 retinae lens side down onto a Whatman® Nuclepore™ Track-Etched Membrane floating on 2 mL of culture medium in a 6-well tissue culture plate and culture at 37°C/5% CO_2_ in a sitting drop of culture medium; 1 retina per drop (Figure 1J).
  h. Re-feed each retina by adding 25 μL culture medium to sitting drop every other day until ready for live imaging.
    ***Note:*** Transfection efficiency can be assessed on standard epifluorescence microscopes, fluorescent protein permitting. In general, latency times between electroporation and visual fluorescent protein detection depend to a large extent, albeit not exclusively, on promoter strength and fluorescent protein brightness. In our experience, when expression of *EGFP* and *DsRed* was driven by a strong promoter (e.g. *CMV*, *CAG* or rod cell-specific *Nrl* promoter (Akimoto et al., 2006)), expression was detectable after 1–2 days and firmly established at 3 days post electroporation.

## Key resources table

**Table:** 

REAGENT or RESOURCE	SOURCE	IDENTIFIER
**Chemicals, peptides, and recombinant proteins**		

DMEM/F-12, HEPES, L-Glutamine	Gibco	Cat#11330057
Penicillin-Streptomycin	Gibco	Cat#15140122
Taurine	Sigma-Aldrich	Cat#T0625
Fetal bovine serum (FBS)	Gibco	Cat#26140079
10× PBS	Gibco	Cat#70011044
Ultrapure dH_2_O (DNAse/RNAse free)	Invitrogen	Cat#11538646
RPMI 1640, phenol red free	Gibco	Cat#11835030
DMEM^gfp^-2 live imaging medium	Evrogen	Cat#MCK02
Paraffin oil	Supelco	Cat#76235

**Critical commercial assays**		

Qiagen plasmid kit (maxi or mega scale)	Qiagen	Cat#12162/12181

**Experimental models: Organisms/strains**		

*Nrl.GFP*^*+/+*^ mice (B6.Cg-Tg(Nrl-EGFP)1Asw/J)	kind gift of A. Swaroop	RRID:IMSR_JAX:02 1232
*Nrl.Cre*^*+/−*^ mice (*C57BL/6J-Tg(Nrl-cre)1Smgc/J*)	The Jackson Laboratory	RRID:IMSR_JAX:02 8941
*Chrnb4.EGFP* mice (Tg(Chrnb4EGFP)CL200Gsat/Mmnc)	MMRRC	RRID:MMRRC_0002 59-UNC

**Recombinant DNA**		

pD10 mU6.TL-shCtrl-TL.shDync1h1 / CMV.FL-pA-FL.EGFP (abbreviated: *shDync1h1/EGFP*)	Aghaizu et al. (2021)	N/A

**Software and algorithms**		

GraphPad Prism	GraphPad	https://www.graphpad.com/scientificsoftware/prism/
Fiji/ImageJ	National Institutes of Health (NIH)	https://imagej.nih.gov/ij/
IMARIS	Bitplane	http://www.bitplane.com/imaris
MATLAB	MathWorks	https://www.mathworks.com/product s/matlab.html
Custom-written Matlab scripts	Aghaizu et al. (2021)	https://github.com/RPearsonLab/Photoreceptor_tracking

**Other**		

Clean bench	N/A	N/A
Tissue culture hood	N/A	N/A
Tissue culture incubator	N/A	N/A
1.0 mL Pasteur Pipette	Starlab	Cat#E1414-1111
Dissection microscope	N/A	N/A
6-well tissue culture plate (sterile)	Thermo Fisher Scientific	Cat#150239
Dumont forceps #5 (2×)	Fisher Scientific	Cat#15483542
Vannas Scissors, Super fine, 8 cm, straight	Fisher Scientific	Cat#50-822-579
30 G microlance 3 needle	BD	Cat#304000
Pulse generator	BTX	Cat#ECM 830
Electroporation cuvette (2 mm gap size)	BTX	Cat#45-0126
Whatman® Nuclepore™ Track-Etched Membranes (0.2 μm, polycarbonate)	Merck	Cat#WHA10417006
Microscopic slides	Fisher Scientific	Cat#11562203
MF nitrocellulose membrane filters	Merck Millipore	Cat#HAWP01300
Ø5.5 cm tissue culture petri dish	VWR	Cat#734-2318
Dow Corning® high-vacuum silicone grease	Sigma-Aldrich	Cat#Z273554
Platinum ring tissue anchor	e.g., Warner Instruments	N/A
Live imaging microscope with 2-photon and environmental control capabilities (temperature, CO_2_)	N/A	N/A

## Materials and equipment

**Table:** DNA solution for electroporation

Reagent	Final concentration	Amount
Plasmid DNA	1 μg/μL	X
PBS (10×)	1×	15 μL
dH_2_O (DNAse/RNAse free)	N/A	Top up to 150 μL
**Total**	**N/A**	**150 μL**

DNA solution was freshly prepared prior to electroporation.

Note that highly concentrated plasmid DNA (ideally > 1 μg/μL) is required and should be produced using Qiagen plasmid kits or similar commercially available kits.

**Table:** Dissection medium

Reagent	Final concentration	Amount
Taurine working solution (50 μM)	150 nM	90 μL
Penicillin-Streptomycin (10,000 U/mL)	10 U/mL	30 μL
DMEM/F-12, HEPES, L-Glutamine	N/A	29.88 mL
**Total**	**N/A**	**30 mL**

Dissection medium was freshly prepared prior to retinal harvest but should be stable at 4°C for 1 week.

**Table:** Culture medium

Reagent	Final concentration	Amount
Fetal bovine serum (100 %)	5 %	0.5 mL
Taurine working solution (50 μM)	150 nM	30 μL
Penicillin-Streptomycin (10,000 U/mL)	10 U/mL	10 μL
DMEM/F-12, HEPES, L-Glutamine	N/A	9.46 mL
**Total**	**N/A**	**10 mL**

Culture medium can be stored at 4°C for 1 week.

**Table:** Live imaging medium

Reagent	Final concentration	Amount
Rutin (100×)	1×	0.1 mL
DMEM^gfp^-2	N/A	9.9 mL
**Total**	**N/A**	**10 mL**

Live imaging medium was freshly prepared prior to imaging.

Note that rutin is supplied as part of DMEM^gfp^-2 kit (Cat#: MCK02).

## Step-by-step method details

### Setting up retina for live imaging

**Timing: 20 min**

This step describes the retrieval of retinae either from living mice or from retinal explant cultures at desired developmental stage to set them up for live imaging. Herein contained are details on retinal dissection, tissue mounting, and live imaging culture set up. Live imaging experiments were performed on an upright confocal microscope; for an inverted microscope the setup would have to be modified accordingly.

**Figure 2:**
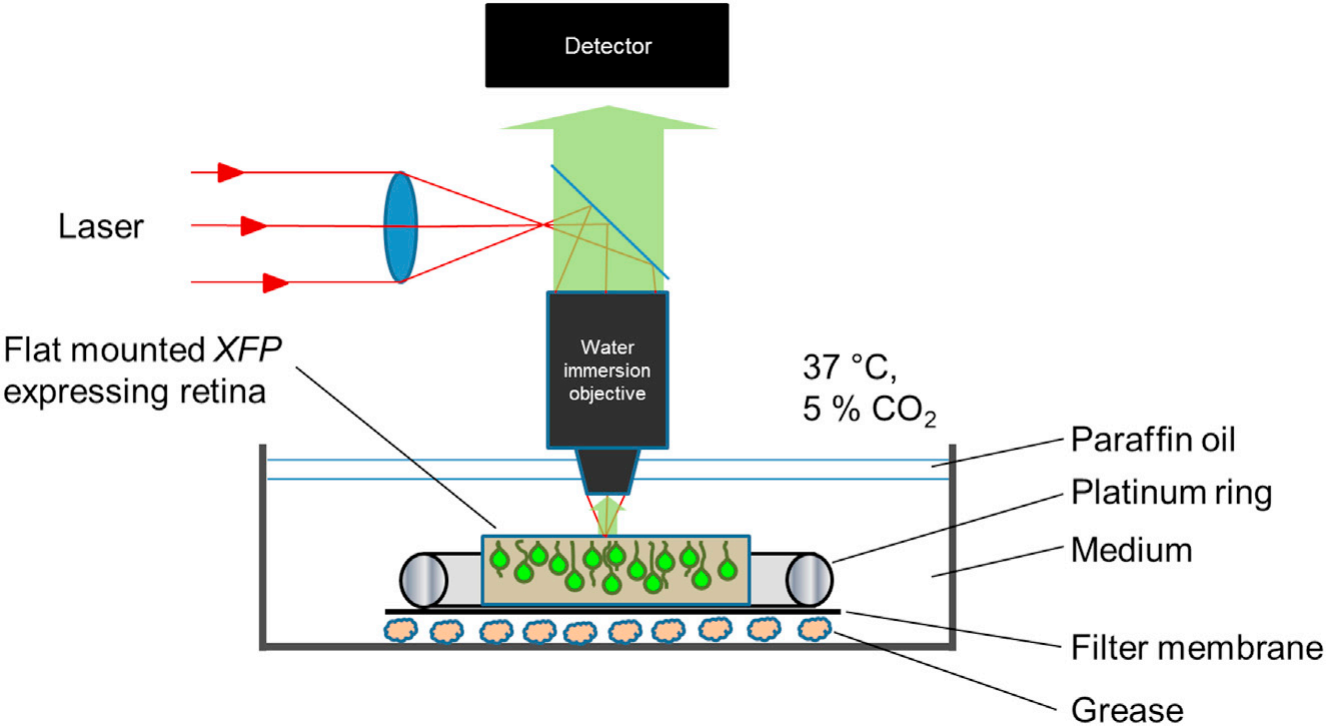
Live imaging setup schematic

1. Retrieval of retina.
  When isolating retinae from live mice or retinal explant cultures, follow steps ‘a.’ or ‘b.’, respectively.
  a. Retrieval from live mice.
    i. Sacrifice mice by cervical dislocation.
    ii. Enucleate eyes and transfer to ice-cold RPMI 1640 medium using forceps (Figure 1A).
    iii. Isolate neural retina (tissue composed of cells of the outer nuclear layer, inner nuclear layer, and ganglion cell layer) by dissection under a dissection microscope in ice-cold RPMI 1640 medium by removing all non-neural tissues (cornea, sclera, iris, choroid, lens, optic nerve, retinal pigment epithelium, and ciliary body) using forceps (Figures 1B and 1C).
      **CRITICAL:** Upon removal of the lens during retinal dissection, ensure that vitreous is removed as well. Failure to remove vitreous can impede the flattening process (also applies to 1.b.ii).
      **CRITICAL:** Care must be taken to remove all pigmented non-neural retinal tissues (retinal pigment epithelium and ciliary body) as the melanin pigment contained in these tissues absorbs the 2-photon infrared light leading to rapid heat-induced tissue damage when the microscope is operated in 2-photon mode (also applied to 1.b.ii).
  b. Retrieval from retinal explant cultures.
    i. Transfer retina from explant culture to ice-cold RPMI 1640 medium.
    ii. Isolate neural retina by dissection under a dissection microscope in ice-cold RPMI 1640 medium by removing lens with forceps.

2. Flattening and mounting retina.
  a. Apply four equidistant radial cuts using fine scissors going halfway towards the centre of the retina. This results in ‘petal’ like retinal segments (Figure 1D).
  b. Transfer retina in a small drop of RPMI 1640 medium onto the edge portion of a microscopy glass slide using a sterile plastic Pasteur pipette with manually widened opening (Figure 1I) such that photoreceptors face the glass slide and retinal ganglion cells face the user (Figure 1D).
  c. Carefully flatten retina onto glass slide by unfolding petals using fine forceps. Petals will resist flattening due to natural curvature of the retina. Flattening is aided by removing some of the RPMI 1640 (Figure 1E).
    **CRITICAL:** Care must be taken to not introduce tissue damage during flattening and unfolding petals. This may be aided by using a clean, fine brush. Even small tissue disruption may result in visible loss of retinal integrity during live imaging.
    **CRITICAL:** (2.c.-3.b.) During these steps, the retina will be exposed to minimal amounts of RPMI 1640 medium at times. Care must be taken to not let the retina dry out.
  d. After successful flattening, remove most of RPMI 1640 medium using lint-free tissue paper.
  e. Immediately place a 0.45 μm MF-Millipore™ nitrocellulose membrane on the retina (with ganglion cell layer touching the membrane) making sure tissue is securely mounted on the membrane (Figure 1F).
  f. Gently transfer membrane-mounted retina back into RPMI 1640 medium by dipping the edge of the glass slide into medium (Figure 1G). The membrane will gradually become visibly hydrated when contacted by the solution, upon completion of which the membrane can be fully transferred using the dissection tools (Figure 1H).

3. Setting up imaging dish for live imaging (Figure 2)
  a. Fix membrane with retina facing up to the bottom of a Ø5.5 cm tissue culture petri dish on a thin layer of vacuum grease.
  b. To ensure secure mounting and to prevent tissue drifting during live imaging, carefully press down membrane onto vacuum grease using blunt tools. Place a platinum ring over the membrane, the added weight of which should further prevent tissue drifting.
  c. Fill the dish with 10 mL DMEM^gfp^-2 live imaging medium.

### Live imaging setup and recording

**Timing: 45 min set up; up to ∼ 12 h time-lapse recording**

The following steps describe setting up the retinal live imaging session (see also Figure 2), at the end of which the user will have generated a time-lapse recording of the fluorescently labelled retinal cells. We routinely used a 2-photon microscope set-up to reduce phototoxicity and to increase photon tissue penetration and *z* resolution.

4. Transfer imaging dish containing flat-mounted retina to microscope environmental chamber pre-equilibrated to 37°C/5% CO_2_.

**CRITICAL:** Allow tissue to acclimatize to 37°C/5% CO_2_ for at least 30 min prior to initiating time-lapse recording to prevent temperature gradient induced tissue drifting.

5. Carefully introduce a water-immersion objective pre-sterilized with 70 % ethanol into the live imaging medium. We routinely used 25× and 40× water-immersion objectives (NA = 0.95 and 0.8 respectively).
6. Affix CO_2_ supplying apparatus to the live imaging dish.
7. Identify suitable field of view to be subjected to time-lapse recording by scanning through microscope eyepiece.
8. Tune the multiphoton laser source to a wavelength suitable for 2 photon-excitation of the fluorescent protein(s) of interest (e.g., 920 nm for EGFP).
9. Further, ensure that laser and detector(s) have been properly set up for signal detection.

***Note:*** In 2-photon mode, the pinhole of confocal microscopes should be fully dilated.

10. Apply a layer of paraffin oil, pre-equilibrated to 37°C, on top of the live imaging medium to prevent medium evaporation during time-lapse recording.
11. Initiate time-lapse recording. In Aghaizu et al. (2021), we acquired *xyzt* image series at a resolution of 512 × 512 pixels, at a step size of 1 μm and at 10 min intervals. Imaging duration frequently reached up to 12 h, although this is critically dependent on starting tissue health/integrity.

**CRITICAL:** Any pigmented cells present within the field of view will absorb infrared light (i.e. 2-photon laser light) and rapidly heat up the tissue, rapidly bringing about tissue destruction.

***Note:*** It is recommended to ensure on occasion that the intended field of view remains in focus; in the event of tissue drift appropriate adjustments are to be made by user.

***Note:*** When imaging photoreceptors, early stages of deteriorating tissue health most frequently presented themselves as individual photoreceptors apically extending out of the outer nuclear layer (see Figure 4A, bottom right panel for an example of apically extruding photoreceptors). Further deterioration usually entails full apical ‘ejection’ of individual and subsequently bigger groups of photoreceptors until tissue integrity is fully compromised.

### *In silico* processing of time-lapse recording and cell tracking

**Timing: minimum 30 mins but can be significantly longer depending on image quality, number of cells to be tracked, and implementation of manual track validation.**

Upon completion of time-lapse recording, generated *xyzt* image series are to be processed and subjected to cell tracking.

**Figure 3:**
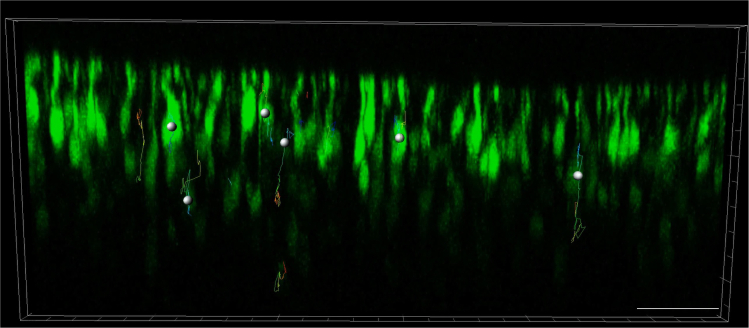
IMARIS tracking of rod somal motility in *xyzt* image stacks obtained from time-lapse recordings This image is a still captured from Methods video S1. Scale bar, 50 μm.

***Note:*** Once set up, cell tracking in the IMARIS spot tracking tool is fully automated. Nonetheless, we recommend manually validating the generated trajectories to avoid including data artifacts into subsequent analysis steps.

12. Process *xyzt* image series in Fiji/ImageJ (Schindelin et al., 2012) with standard imaging processing tools available, such as noise reduction, to achieve maximum possible image quality and signal to noise ratio. Ensure that processing steps are kept consistent.
13. Register *xyzt* image series (*in silico* correction of tissue drift), as some amount of tissue drifting during time-lapse recording is common. This can be accomplished in Fiji/ImageJ either with the ‘Correct 3D drift’ plug-in (Parslow et al., 2014) for automated drift correction based on automated landmark recognition or the ‘Descriptor-based series registration (2D/3D + t)’ plug-in (Preibisch et al., 2010) in cases where automated registration failed and registration landmarks had to be defined manually.
14. Export registered *xyzt* image series as ∗.tif file.
15. Perform cell tracking using software of choice. In Aghaizu et al. (2021) we used the spot tracking tool within IMARIS software. IMARIS software is able to identify rod cell bodies based on the preceding input of size and location parameters (Methods video S1; video still shown in Figure 3). Rod somata were typically ellipsoid in shape, measuring ∼ 5 μm along the short (lateral) and ∼ 7.5 μm along the long (radial) axis and are typically located deeper within the tissue compared to the developing bulbous segment structures.

16. Export completed cell tracking data as ∗.xls file.

Methods video S1. IMARIS tracking methodology (see also Figure 2; related to step 15)

### Data processing and analysis

The primary data outputs from the processing and analysis steps will be average mean squared displacement (AvMSD) as well as instantaneous velocity and instantaneous acceleration values for the population of cells analysed. In Aghaizu et al. (2021), these were generated by importing IMARIS-generated tracking information into Matlab followed by execution of custom-written Matlab scripts (available on the project Github repository on https://github.com/RPearsonLab/Photoreceptor_tracking).

17. Arrange IMARIS-generated tracking data in Microsoft Excel according to Table 1. ‘#’ denotes numbered data point ID in Excel; ‘Trajectory ID’ denotes unique numbered identifier of individual trajectories; ‘Time index’ denotes time point within time-lapse recording, at which data point was generated (note that this is in integer rather than time units); ‘Position *x/y/z*’ denotes 3D positional information.

**Table 1:** Example trajectory data

#	Trajectory ID	Time index	Position *x*	Position *y*	Position *z*
1	1	1	1.0	1.0	60.0
2	1	2	1.0	1.0	59.0
3	1	3	1.0	1.0	54.0
4	1	4	1.0	1.0	51.0
5	1	5	1.0	1.0	45.0
6	2	1	1.0	1.0	30.0
7	2	2	1.0	1.0	30.0
8	2	3	1.0	1.0	30.0
9	2	4	1.0	1.0	30.0
10	2	5	1.0	1.0	30.0
11	3	1	1.0	1.0	10.0
12	3	2	1.0	1.0	10.1
13	3	3	1.0	1.0	10.5
14	3	4	1.0	1.0	10.9
15	3	5	1.0	1.0	11.0

***Note:*** Matlab scripts were designed to read-in tracking data in the format specified in Table 1. The user is advised to either manipulate data to match Table 1 or to modify Matlab script accordingly.

18. Open and run the Matlab script 'MSDforztrackingdata.m' (see Github project repository) to read-in trajectory data and to calculate AvMSD & standard error values. Users will be prompted to navigate to the source trajectory file upon initiation of the script.

AvMSD will be calculated according to the equation

$$MSD\left(\Delta t\right)=\;\frac{1}{N-n}\sum_{i=1}^{N-n}{\left[z\left(\left(i+n\right)\delta t\right)-z\left(i\delta t\right)\right]}^{2}$$

where z(iδt) and z((i+n)δt) are the z positions of a given nucleus at time frames iδt and (i+n)δt respectively, n is an integer representing the time interval between those positions and N is the total number of time points within the time-lapse recording. The only script manipulation required to run 'MSDforztrackingdata.m' is to specify the range of trajectory IDs to be analysed from source file (e.g.,: 1, 2, 3,…, n); line 134.

group = [1, 2, 3];

***Note:*** The above formula calculates the MSD at all possible time lags and produces averages within individual trajectories as well as the entire group of specified trajectories. For instance, at a time lag of 2 according to Table 1, all possible MSDs between time indices 1 & 3, 2 & 4, and 3 & 5 for trajectories 1–3 are calculated and averaged to produce the AvMSD value at time lag 2.

19. Upon completion of script 'MSDforztrackingdata.m', the Matlab will have generated result outputs in Vector/matrix format denoted ‘AvMSD’ and ‘standarderrors’ containing AvMSD and standard error values with increasing time lag.

***Note:*** For our cell type of interest (photoreceptors), motility occurred predominantly along the *z* axis (radial) rather than in *xy* (lateral) directions. AvMSD and instantaneous velocity calculations in the Matlab script are thus based exclusively on *z* motility and the user is advised to adjust the Matlab script accordingly for higher dimensional analyses.

20. Open and run 'ztrajectory_gen.m' to convert read-in trajectory data stored in array format into matrix 'ztrajectories'. Individual trajectories are listed by row.

lengths = ones(length(trajectories),1);

for i = 1:length(trajectories)

 lengths(i) = length(trajectories{i});

end

t = max(lengths);

ztrajectories = zeros(length(trajectories),t);

for i = 1:length(trajectories)

 if trajectories{i}(1,2) > 1

 leading = NaN∗ones(length(1:(trajectories{i}(1,2)-1)),1);

 else

 leading = [];

 end

 if trajectories{i}(end,2) < t

 trailing = NaN∗ones(length(trajectories{i}(end,2):t-1),1);

 else

 trailing = [];

 end

 ztrajectories(i,:) = [leading;trajectories{i}(:,5);trailing];

end

21. Open and run 'Trajectory_analysis.m' for further kinetic analyses (instantaneous velocity, instantaneous acceleration) of trajectory data generated in (17.) and (18.). Upon completion, the Matlab script will have generated result outputs in matrix format denoted ‘dfff2’ and ‘d2fff2’ containing smoothened instantaneous velocity and acceleration values respectively. ‘dyyy’ contains un-smoothened instantaneous velocity data.

ddd = ztrajectories;

ncells = size(ddd,1);

nsamples = size(ddd,2);

xxx = nan(ncells,nsamples);

yyy = nan(ncells,nsamples);

for icell = 1:ncells

 x = find(isfinite(ddd(icell,:)));

 y = ddd(icell,x);

 xq = min(x):max(x);

 yq = interp1(x,y,xq,'linear');

 xxx(icell,xq) = xq;

 yyy(icell,xq) = yq;

end

fff = filter( [1 1 1]/3, 1, yyy' )';

fff(:,[1:3 end-2:end]) = NaN;

fff = circshift(fff,[0 -1]);

dyyy = diff(yyy,1,2)./10;

z = nan(ncells,1);

dyyy = horzcat(dyyy,z);

dfff = diff(fff,1,2)./10;

z = nan(ncells,1);

dfff = horzcat(dfff,z);

windowsize = 4;

b = (1/windowsize)∗ones(1,windowsize);

a = 1;

dim = 2;

dfff2 = filter(b,a,dfff,[],dim);

dfff2 = circshift(dfff2,[0 -1]);

d2fff = diff(dfff,1,2);

z = nan(ncells,1);

d2fff = horzcat(d2fff,z);

windowsize = 4;

b = (1/windowsize)∗ones(1,windowsize);

a = 1;

dim = 2; % to filter rows, not columns

d2fff2 = filter(b,a,d2fff,[],dim);

d2fff2 = circshift(d2fff2,[0 -1]);

***Note:*** Matlab script was custom written for time-lapse recordings acquired at 10 min intervals. This is reflected in instantaneous velocity calculations, where velocity between consecutive frames (μm/10 min) needed to be divided by 10 to obtain instantaneous velocity (μm/min) (lines 59 and 64 in 'Trajectory_analysis.m').

22. Plot and interpret data generated in (17.) and (19.) in software of choice, e.g., Matlab or Graphpad Prism.
  a. Plot AvMSD (± standard errors) vs time lag (e.g., 0, 10, 20, 30,…n min). Depending on the nature of motility, AvMSD vs time lag curves will be either linear or curved.
    i. A linear AvMSD vs time lag curve is produced if random/non-directional motion dominates in the population of analysed cells. Here, the MSD is a linear function of elapsed time Δt,
      MSD=2RDΔt
      with a one-dimensional slope of 2R*D = 2D*, where R is the dimensionality (in the present study, R = 1) and where *D* is the coefficient of movement. Apply linear function curve fitting to obtain the slope ‘2D’ and subsequently calculate ‘D’ to quantitatively compare AvMSD steepness.
    ii. Directed motions in the analysed cell population result in AvMSD vs time lag curves with positive curvature. Here, the MSD displays a quadratic dependence on elapsed time. Apply quadratic function curve fitting
      $$y=ax^{2}$$
      to obtain the quadratic factor ‘a’ as numerical representation of curve steepness.
  b. Plot instantaneous velocity and acceleration values as histograms to show frequency distributions.

## Expected outcomes

**Figure 4:**
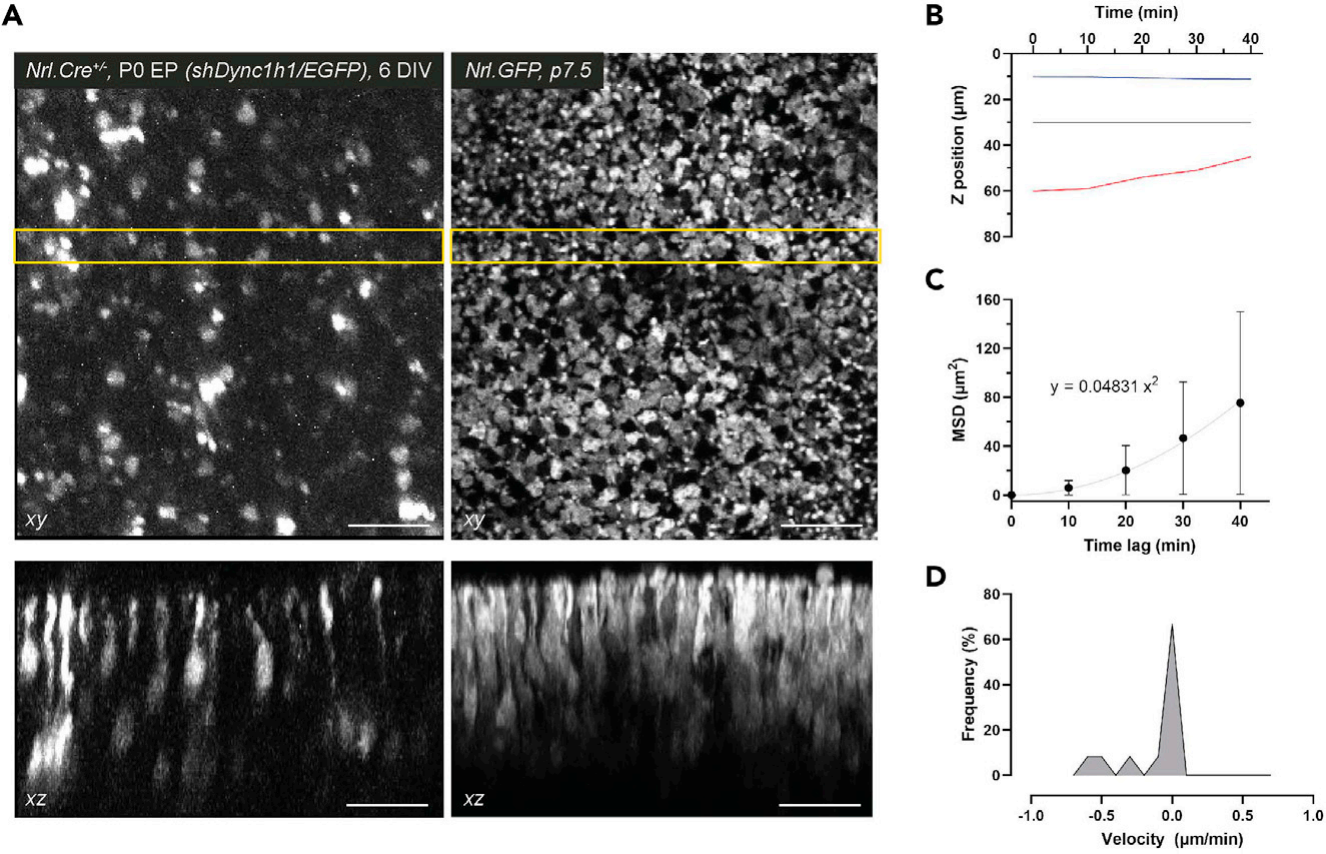
*z* motility quantification of rod photoreceptors in *xyzt* image stacks (A) top-down (*xy*) and orthogonal views (*xz*) of live imaged retinae with rod photoreceptors labeled either conditionally by electroporation (left panels; *Nrl.Cre*^*+/−*^ retina, electroporated at P0.5 with *shDync1h1/EGFP* and cultured for 6 DIV) or by transgenic reporter labeling (right panels; *Nrl.GFP*, P7.5). (B) Example trajectory data *z* positions. (C) Example trajectory data AvMSD vs. time lag plot including quadratic function curve fitting. Error bars show SEM. (D) Example trajectory data instantaneous velocity histogram. Scale bars, 25 μm.

Following successful live imaging the user will have generated a *xyzt* image stack. The fluorescence signal distribution within the tissue will depend on the exact experimental design. For reference, we provide two examples where rod photoreceptors were labeled using different approaches (Figure 4A): *Nrl.Cre*^*+/−*^ retina, electroporated at P0.5 with *shDync1h1/EGFP* and cultured for 6 days *in vitro* (DIV) before live imaging (left panels); *Nrl.GFP*, postnatal day (P) 7.5 (right panels). Rod somal position can then be tracked as described in step 15. Top-down (*xy*) and orthogonal views (*xz;* maximum z projection of 5 adjacent slices at 1 μm interval) of the image stacks are shown. For the purpose of this protocol, the presented quantifications are based on the example trajectory data provided in Table 1.

Changes in *z* position over time were plotted for the example trajectory data (Figure 4B). Average mean squared displacement and standard error values were obtained using the provided Matlab script (step 18.) and plotted versus time lag (Figure 4C). Note the positive curvature in the data points, due to which quadratic function curve fitting (y = ax^2^) should be applied to obtain the quadratic coefficient. Where average MSD data points follow a straight line, linear curve fitting should be applied, in order to obtain the coefficient of movement according to step 22.a.i. Instantaneous velocity values were derived using the provided Matlab script (steps 21, 22.) and plotted as a histogram (Figure 4D).

## Limitations

The protocol described here is inherently low throughput as only one retina can be live imaged at a time. This bottleneck will be especially evident when time-lapse recordings are conducted for ∼ 12 h.

*In silico* trajectory verification presents another potential bottleneck in the protocol as we recommend verifying each individually generated trajectory. Generally, trajectory generation reliability increases with higher signal-to-noise ratios in the *xyzt* image series. Producing high-quality time-lapse recordings as well as appropriate image processing workflows are thus essential to increasing the data output.

## Troubleshooting

### Problem 1

Step 1.a.: Neural retina prolapse from eyeball during its isolation resulting in retinal tissue damage.

### Potential solution

During eyeball dissection to isolate neural retina, the eyeball needs to be carefully opened using dissection tools as described. Small amounts of pressure applied to the eyeball during this step can lead to the neural retina prolapsing from introduced eyeball openings, compromising retinal integrity. This can be avoided by piercing the eyeball at the interface between cornea and sclera using a fine gauge needle (e.g., 30 G BD microlance 3 needle, Cat#304000).

### Problem 2

Step 11: tissue drift during time-lapse recording.

### Potential solution

In case *xyz* tissue drift is detected during time-lapse recording, adjust field of view and/or upper/lower limits of z-stack accordingly in the intervals between time series points (this may require prematurely stopping the acquisition process and initiating a new *xyzt* acquisition, which can subsequently be concatenated *in silico* with the initial acquisition, for instance using Fiji/ImageJ). To avoid tissue drift in future experiments, consider the following options during tissue mounting (steps 2. and 3. in step-by-step method details):

Using a heavier platinum harp.

More firmly pressing the edges of the nitrocellulose membrane with mounted retina onto the vaccume grease layer using a round edge of curved forceps.

Securing the edge of nitrocellulose membrane with mounted retina with extra vacuum grease contiguously spread over both the nitrocellulose edge and the underlying petri dish.

### Problem 3

Step 11: signs of boiling/bubbling evident during time-lapse recording.

### Potential solution

Boiling and bubbling are usually a consequence of pigmented cells (usually black in colour) present within the field of view. These absorb infrared light and rapidly heat up the tissue leading to irreversible tissue damage. To prevent this from occurring, consider imaging a field of view completely devoid of pigmented cells or completely removing all pigmented tissues/cells from neural retina during relevant dissection steps and/or prior to live imaging.

### Problem 4

Step 11: loss of retinal integrity during time-lapse recording.

### Potential solution

Even the most benign tissue damage introduced during retinal dissection and mounting tends to compound during live imaging, eventually leading to loss of retinal integrity. This can be mitigated by careful tissue handling and/or by avoiding damaged areas of the retina during live imaging.

### Problem 5

Step 15: Spot tracking in IMARIS produces too many tracking artifacts, misrepresenting true somal movements.

### Potential solution

Using an optimized workflow in Fiji/ImageJ can contribute greatly towards generating higher quality, high signal-to-noise time-lapse recordings post acquisition, which can improve subsequent tracking in IMARIS. While there is no ‘one-size-fits-all’ approach applicable to all time-lapse experiments, we have found the ‘Remove outliers’ tool in Fiji/ImageJ particularly useful to remove noise artifacts that can impede faithful tracking in IMARIS. Set parameters to remove “bright” outliers at a radius and threshold that approximately match the characteristics of noise artifacts.

Furthermore, the spot detection tool in IMARIS offers filter options to remove artifactual trajectories. The ‘Quality’ filter can be utilized to retain only high-quality tracking points, rather than lower-quality tracking points, which are more likely to be associated with imaging noise and/or background signal. Other IMARIS filters that proved to be useful for tracking photoreceptor somata that primarily exhibit radial (along the *z* axis) motility were *x* and *y* dimension filters. These can be used to restrict the maximum permissible amount of tangential (*xy*) motility in the generated tracks, which further eliminated artifactual tracking in our experiments.

## Resource availability

### Lead contact

Further information and requests for resources and reagents should be directed to and will be fulfilled by the lead contact, Rachael Pearson (rachael.pearson@kcl.ac.uk).

### Materials availability

This study did not generate new unique reagents.

## Data Availability

The data sets generated during and/or analysed during the current study are available from the lead author on request. The code generated during this study is available on the project Github repository on https://github.com/RPearsonLab/Photoreceptor_tracking.
